# Dying in self-defence: a comparative overview of immunogenic cell death signalling in animals and plants

**DOI:** 10.1038/s41418-022-01060-6

**Published:** 2022-10-04

**Authors:** Takaki Maekawa, Hamid Kashkar, Núria S. Coll

**Affiliations:** 1 Department of Biology, Institute for Plant Sciences, University of Cologne, 50674 Cologne, Germany; 2 CEPLAS Cluster of Excellence on Plant Sciences at the University of Cologne, Cologne, Germany; 3 Faculty of Medicine and University Hospital of Cologne, Institute for Molecular Immunology, University of Cologne, 50931 Cologne, Germany; 4 Faculty of Medicine and University Hospital of Cologne, Center for Molecular Medicine Cologne (CMMC), University of Cologne, 50931 Cologne, Germany; 5 Cologne Excellence Cluster on Cellular Stress Responses in Aging-Associated Diseases (CECAD), University of Cologne, 50931 Cologne, Germany; 6 Centre for Research in Agricultural Genomics (CRAG), CSIC-IRTA-UAB-UB, Campus UAB, 08193 Bellaterra, Spain; 7 Consejo Superior de Investigaciones Científicas (CSIC), 08001 Barcelona, Spain

**Keywords:** Immune cell death, Microbiology

## Abstract

Host organisms utilise a range of genetically encoded cell death programmes in response to pathogen challenge. Host cell death can restrict pathogen proliferation by depleting their replicative niche and at the same time dying cells can alert neighbouring cells to prepare environmental conditions favouring future pathogen attacks. As expected, many pathogenic microbes have strategies to subvert host cell death to promote their virulence. The structural and lifestyle differences between animals and plants have been anticipated to shape very different host defence mechanisms. However, an emerging body of evidence indicates that several components of the host–pathogen interaction machinery are shared between the two major branches of eukaryotic life. Many proteins involved in cell death execution or cell death-associated immunity in plants and animals exert direct effects on endomembrane and loss of membrane integrity has been proposed to explain the potential immunogenicity of dying cells. In this review we aim to provide a comparative view on how cell death processes are linked to anti-microbial defence mechanisms in plants and animals and how pathogens interfere with these cell death programmes. In comparison to the several well-defined cell death programmes in animals, immunogenic cell death in plant defence is broadly defined as the hypersensitive response. Our comparative overview may help discerning whether specific types of immunogenic cell death exist in plants, and correspondingly, it may provide new hints for previously undiscovered cell death mechanism in animals.

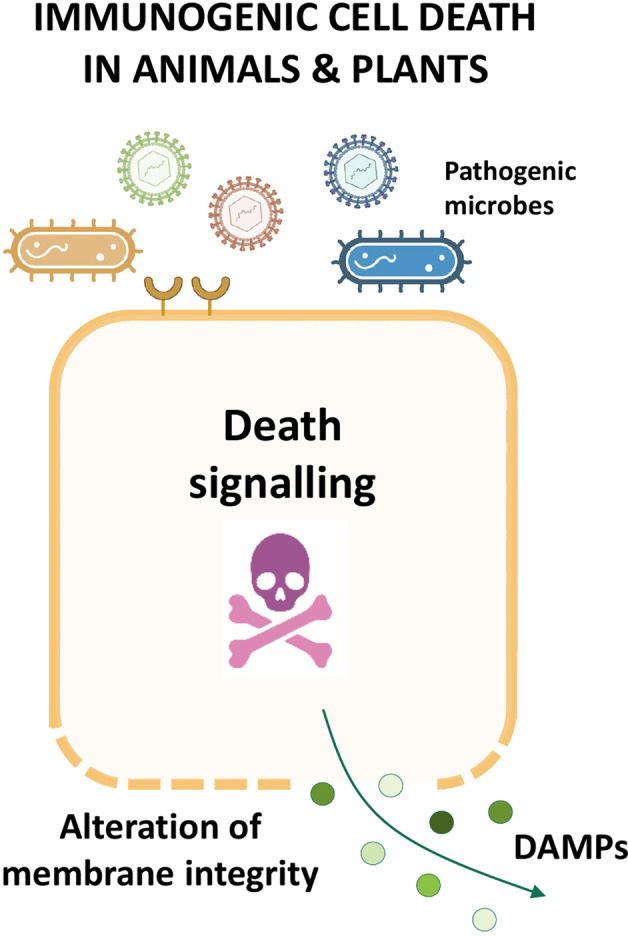

## Facts

- Cell death is a fundamental immune defence mechanism in animals and plants.
- Several components of cell death machinery are shared between animals and plants.
- Alteration of endomembrane integrity represents a crucial step in immunogenic cell death signalling in animals and plants.
- Pathogenic microbes have evolved strategies to subvert host cell death in animals and plants to promote their virulence.

## Open questions

- Caspases are increasingly anticipated as a molecular switch in cell death and cellular signalling. What is the evolutionary conserved function of cell death proteases in animals and plants?
- Lytic cell death and membrane disintegration are viewed as an initiator of neighbouring responses and systemic immunity in animals. How does cell death in plants involve neighbouring cells and provoke systemic immunity?
- How does limited/controlled membrane disruption control cell death, neighbouring responses and pathogen progression in animals and plants?
- Are there common molecular structures that are released from animal and plant cells and how do they impact immunity and pathogen progression?
- Does plant hypersensitive cell death resemble the distinct features of regulated necroptosis and/or pyroptosis in animals?
- How does the increase of cation concentration in the cytosol caused by the action of plant resistosomes translate into immunogenic cell death in plants?

## Introduction

Cell death represents a common and fundamental process in host-microbe interactions in both plants and animals [1, 2]. Not surprisingly, plant and animal pathogens have evolved various means to specifically avoid or subvert host cell death as part of their virulence strategies. Such co-evolutionary struggles between hosts and pathogens have resulted in some of the most complex and interesting biological interactions [3]. The structural and lifestyle differences between plants and animals and their respective pathogens have been expected to involve very different defence mechanisms and pathogenic strategies. However, accumulating evidence indicate that several components of host–pathogen interactions are shared between the two phyla.

**Fig. 1:**
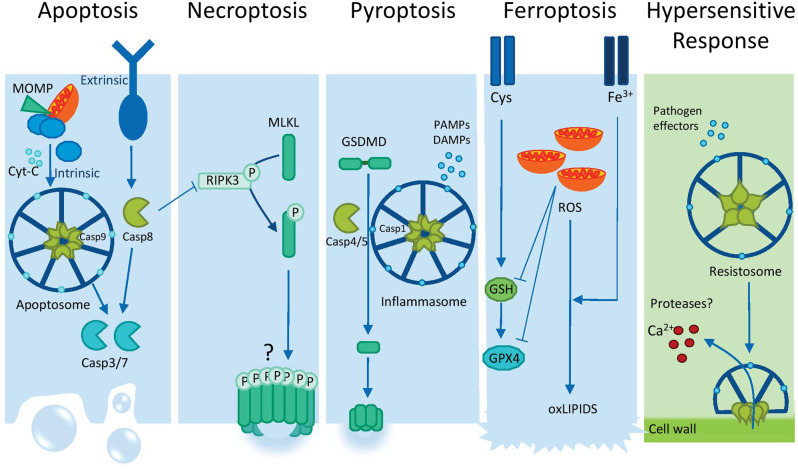
Schematic comparison of molecular cell death features of animal and plant cell death types. Schematic display of cell death modalities of animals (apoptosis, necroptosis, pyroptosis and ferroptosis, in blue) and plants (hypersensitive response, green), highlighting the main regulators, formation of “deathosome” structures and impact on membrane integrity. Plant TNL resistosomes are not included, since the scheme mostly focuses on the impact of immune cell death on plasma membrane.

The death of pathogen-infected cells can have beneficial or detrimental consequences for the host. Host cell death can restrict pathogen proliferation by destroying their replicative niche. Alternatively, pathogens can induce host cell death to disseminate and infect adjacent tissues. Beyond this categorical view of cell death, the quality of cell death or how exactly cells die is emerging as a central determinant of the fate of the affected tissues. Alteration of membrane integrity represents one of the key steps outlining a model that the immune system is more concerned with entities that do damage rather than with those that are only foreign [4]. The concept of damage-associated molecular patterns (DAMPs) has been originally proposed to explain the potential immunogenicity of dying cells and immunogenic cell death describes cell death modalities that stimulate an immune response against dead cell-antigens [5, 6]. Accordingly, a number of genetically controlled and molecularly defined cellular death processes that utilise distinct cellular death machinery and yield different tissue responses have been characterized in animal cells, including apoptosis, necroptosis, pyroptosis and ferroptosis (Fig. 1 and Table 1). Furthermore, accumulating evidence suggests that different cell death processes are deeply interlinked, serve as backup mechanisms and thereby guarantee cellular death to mount immunity.

**Table 1:** Comparison of the major cell death processes between animals (blue) and plants (green).

“?” and “-” indicate “potentially, but not yet fully elucidated” and “not detected”, respectively.

^a^Ferroptosis-like cell death has been described in plants (see text for more details), although molecular details remain limited.

^b^The hypersensitive response (HR) can be considered “inflammatory”, as HR releases immunogenic molecules to the extracellular space.

In plants, immunogenic cell death has been broadly termed hypersensitive response (HR). HR involves generation of reactive oxygen species (ROS) and elevation of intracellular Ca^2+^ levels (Fig. 1) and is often caused by activation of members of the intracellular nucleotide-binding domain leucine-rich repeat containing (NLR) receptor family [7–9]. The amplitude of HR can be intensified upon recognition of conserved pathogen-associated molecular patterns (PAMPs) by plasma membrane resident receptors (PRRs) [10, 11]. Because NLR-triggered immunity also intensifies PRR-triggered immunity [10, 11], this mutual potentiation mechanism between NLR and PRR signalling acts as an amplification step in immune signalling and HR. Whether specific types of immunogenic cell death exist in plants under the wide umbrella of HR remains to be clarified.

Despite the differences in nomenclature, both plant and animal pathogens can be classified as killers or non-killers, as previously proposed [12]. This simplistic view is useful when comparing host–pathogen interactions in animals and plants. Essentially, non-killer pathogens feed on living tissue and need to maintain a living host, whereas killer pathogens feed on dead or dying tissue. As a consequence, non-killer pathogens have evolved a variety of mechanisms to block host cell death, while in turn, killer pathogens can induce cell death in the host for their own benefit. Naturally, intermediate situations exist, whereby pathogens can switch from non-killers to killers during their life cycle.

In fact, suppression of cell death by certain pathogens as a virulence strategy may have acted as an evolutionary driving force for the host to evolve different cell death modalities [13, 14]. Furthermore, it is increasingly evident that the pathogenesis of microbial infection does not only rely on the host cell death as a final event, but also depends on the mode of cell death and the quality of the intercellular communication within the infected tissue. These intercellular communications do not only activate host defence mechanisms, but also boost tissue regeneration to achieve full resolution of the infection. On the other hand, successful pathogens are able to thwart this process and cause overwhelming tissue damage that supports the propagation and spread of the pathogen.

In the sections below we provide detailed descriptions of the death processes linked to microbial infection in animals and plants.

### Regulation of host–pathogen interaction by apoptosis

Apoptosis is the best studied form of animal cell death and is morphologically characterized by the ordered disassembly of the dying cell within the boundaries of an intact plasma membrane. Apoptosis is controlled by cysteine-dependent aspartate-specific proteases termed caspases. With the exception of caspase-1—enriched in monocytes/macrophages, and caspase-14—restricted to keratinocytes, caspases are widely expressed as cytosolic/nucleoplasmic zymogens [15]. Once activated by an upstream stimulus, caspases perform limited proteolysis resulting in inactivation or activation of distinct downstream cascade of signalling events permitting the controlled demolition of cells. Within the family, apoptotic caspases are distinguished as initiator or executioner caspases. The initiator caspases translate upstream death signals into proteolytic action upon association with multicomponent signalling complexes which generate an active protease [16]. Executioner caspases can be activated by initiator caspases and subsequently convert the proteolytic action into an apoptotic phenotype by processing distinct downstream substrates. The main pathways for caspase cascade activation are the extrinsic and intrinsic pathways. In the extrinsic pathway, the activation of cell surface death receptor leads to the formation of a protein complex including the initiator caspase-8 [17]. In the intrinsic/mitochondrial pathway the initiator caspase-9 is activated after mitochondrial outer membrane permeabilization (MOMP) and the release of cytochrome c. Once in the cytosol, cytochrome c induces the formation of a cytosolic protein complex––the apoptosome—consisting of the apoptotic protease activating factor 1 (Apaf-1) [18] and caspase-9. Both caspase-8 and −9 proteolytically activate executioner caspase-3 and -7 which ultimately drive the characteristic morphology of apoptosis including membrane blebbing, chromosomal DNA fragmentation, packaging of cell constituents into apoptotic bodies and eventually cell death.

Pathogens, particularly when growing inside a host cell, often activate a plethora of cellular stress responses that are normally sensed by apoptotic machinery and translated into proteolytic action involving caspases. Inhibition of apoptosis allows pathogens to optimize replication and progeny synthesis by prolonging the infected cell life. Accordingly, caspases represent an important molecular target for pathogens to control apoptosis. This is particularly the case for viral pathogens, as they are obligate and intracellular pathogens, therefore they have evolved multiple strategies to block caspase activity, as shown with the cytokine response modifier A (CrmA) from cowpox virus [19–21], p35 from baculoviruses [22, 23] or FLICE/caspase-8-inhibitory proteins (v-FLIPs) from gamma-herpesviruses and the tumorigenic human molluscipoxvirus [24]. Other anti-apoptotic viral gene products have been shown to inhibit apoptosis without inhibiting caspase activity, such as inhibitor of apoptosis protein (IAP) from baculoviruses [25]. A number of different viral pathogens including *Adenoviridae*, *Birnaviridae*, *Herpesviridae* and *Poxviridae* express viral orthologues of Bcl-2, a key regulator of MOMP, efficiently controlling intrinsic apoptosis during the course of viral infection [26].

In contrast to viruses, only a minority of bacterial pathogens propagate within the cytosol and have direct access to caspases. Instead, they usually reside and multiply outside the cell or within intracellular vacuoles and deliver their effector proteins into their hosts’ cytosol via secretion systems [27]. Regardless, inhibition of apoptosis plays an integral role in bacterial pathogenesis at early stages of infection, and bacteria utilise a number of different strategies for the inhibition of host cell apoptosis [28]. These include caspase inhibition, as it has been shown for *Rickettsia rickettsii*, *Shigella flexneri* and *Crassostrea gigas* [29–32] and upon lipopolysaccharide (LPS) direct binding to the executioner caspase-3 [31, 32]. Furthermore, *Escherichia coli* inhibits caspase activation by N-linked glycosylation of the components of the death receptor signalling complex [33]. Intrinsic/mitocondrial apoptosis is also controlled by many bacterial pathogens including *Chlamydia* [34, 35], *Mycobacterium tuberculosis* [36], and *Coxiella burnetii* [37] by interfering with the expression or degradation of different members of the Bcl-2 protein family.

Although initially and extensively studied in the context of apoptosis, caspases are increasingly considered as versatile molecular switch controlling different cellular outcomes. In particular, inhibition of caspase-8 not only fails to block cell death, but also causes inflammatory lytic cell death by involving necroptosis or pyroptosis (see below) [13, 14]. Lack of caspase-9 activity indeed uncouples the apoptotic death from mitochondrial damage, but it engages various inflammatory signalling and leads to lytic cell death [38]. Accordingly, inhibition of caspase activity by pathogens may not solely increase the lifespan of the host cell, it rather changes the nature of death and may cause beneficial or detrimental tissue responses which deserves further consideration during the course of host–pathogen interaction.

In plants, there is no apoptosis. As outlined above, plant pathogens can trigger HR, a plant-specific type of regulated cell death that involves activation of proteolytic enzymes [39]. As we discuss in this review, several exciting similarities between HR and different forms of regulated lytic cell death (pyroptosis, necroptosis) are emerging, especially at the molecular level. However, considering HR as a form of apoptotic-like cell death is a very limiting misnomer often used in the literature that leads to confusion and should be avoided.

Proteases are probably one of the most ancient families of enzymes [40]. However, plants do not encode caspases in their genomes [41]. Although caspase-like activities have been shown to be important for HR, these are carried out by proteases with little structural resemblance to caspases [39]. In turn, metacaspases, which belong to the same superfamily as caspases and share certain structural similarities with them, do not cleave after aspartate residues (positively charged), but rather lysine or arginine (negatively charged) and therefore they cannot be considered caspases [42]. Metacaspases have been shown to be important for HR regulation [43], although their mode of action in this process remains unclear. Interestingly, available crystal structures suggest that functional metacaspases act as monomers [44, 45].

### “Deathosomes”, macromolecular apparatuses causing cell death

#### Inflammasome-initiated pyroptosis in animal cells

Inflammasomes are multiprotein complexes that are formed upon activation of PRRs following the detection of PAMPs or DAMPs in the cytosol of host cells [46]. Inflammasomes serve as platforms for the activation of caspase-1 which promotes the proteolytic maturation of the cytokines interleukin-1β (IL-1β) and IL-18. Caspase-1 additionally cleaves gasdermin D (GSDMD) unleashing its pore-forming domain and inducing a lytic type of cell death that is known as pyroptosis.

Although initially described as caspase-1–dependent cell death, pyroptosis can be additionally induced by caspase-4 (or its orthologues caspase-11 in mouse) and caspase-5 each of which cleaves GSDMD and induces membrane pores [47, 48] (Fig. 1). Activation of caspase-1 requires the formation of an inflammasome which is initiated upon pattern recognition by NLRs (Nlrp3, Nlrp1b and Nlrc4), the cytosolic DNA sensor AIM2 (absent in melanoma 2) and Pyrin, which subsequently recruits caspase-1 zymogen directly or through the adaptor protein ASC (apoptosis-associated speck-like protein containing a CARD) [49]. Caspase-4, −5 and −11 are activated by the detection of cytosolic LPS and do not require the formation of canonical inflammasomes [50]. Unlike caspase-1, −4, −5 and −11 are not able to process IL-1β and IL-18.

Pathogens manipulate various aspects of pyroptosis to escape the immediate and efficient killing of the host cells. *Yersinia* outer protein K (YopK) and M (YopM) block the activation of the inflammasome [51, 52]. *Pseudomonas aeruginosa* effectors C120HSL, ExoU and ExoS inhibit NLRC4, NLRP3 and caspase-1 [53, 54]. Bacterial pathogens also efficiently manipulate caspase-4, −5 and −11-mediated pyroptosis. *Francisella* and *Shigella* reshape their LPS and escape efficient cytosolic detection by caspase-4, −5 and −11 [55, 56]. *Shigella* OspC3 effector can efficiently inhibit caspase-4 and −11 by direct binding or post-translational modification [57, 58]. Caspase-1 can also be inhibited by viruses such as poxvirus serpins [19]. Poxviruses also express pyrin-only proteins that inhibit inflammasome activation by direct binding ASC and NLRs [59]. Papillomaviruses inhibit pyroptosis by inducing proteosomal degradation of the inflammasome [60]. Kaposi’s sarcoma-associated herpesviruses express the NLRP1 homolog Orf63, which subverts the function of NLRs [61]. Measles virus and influenza virus inhibit the NLRP3 inflammasome [62, 63]. Enterovirus 71 inhibits pyroptosis by cleaving and inactivating GSDMD [64].

GSDM-like proteins have been identified in taxa outside of animals such as fungi and bacteria [62]. Fungal and bacterial GSDM-like proteins share many features with mammalian GSDMs: they are activated by proteases and they have the ability to form pores and permeabilize plasma membranes mediating cell death upon allorecognition and upon phage infection, respectively [63, 64]. These observations establish GSDMs as an extremely ancient mechanism of cell death and membrane pore formation, which may be important for the understanding of the evolution of regulated necrosis in immunity. Currently, no GSDM-like proteins have been identified in plants. Further structural and functional analyses will be instrumental for identifying GSDM-like proteins in plants, paving the way for a better understanding of cell death in these organisms.

#### Resistosome-initiated cell death in plants

In the last few years, a major breakthrough in plant immunity has been the identification of supramolecular complexes assembled upon NLR activation with a key role in defence, which have been dubbed “resistosomes” [65]. In plants, NLRs are broadly classified according to their N-terminal domain as coiled-coil (CC) domain NLRs (CNLs) or TOLL/interleukin 1 receptor (TIR)-like domain NLRs (TNLs). Additionally, a subclass of CNLs has been identified as RPW8-like CC domain containing NLRs (RNLs) [66–68]. The RPW8-like CC domain is alternatively called the HeLo-domain due to a characteristic N-terminal four-helix bundle structure (also see below) [69, 70]. As TNL-mediated HR and immunity require RNLs, RNLs are also known as “helper-NLR” [68]. Structural and biochemical data on resistosomes have helped understanding the differences of CNL- and TNL-RNL pathways and their mechanistic similarities to animal immune deathosomes. Structurally, CNL resistosomes resemble the wheel-like inflammasome comprising animal NLRs [71, 72].

In the context of the two-tiered plant immune system [73], it has been proposed that initial PAMP recognition by surface receptors triggers a fast and transient elevation of cytosolic Ca^2+^ concentration (i.e, Ca^2+^ influx), whereas NLR activation leads to a long-lasting Ca^2+^ influx that would result in cell death, overcoming potential pathogen-mediated immune suppression [74]. The spatiotemporal pattern of Ca^2+^ spikes upon NLR activation at a single cell level as well as at tissue-level might be distinctive to activate cell death-executing proteins. In addition, Ca^2+^ could directly act as an activator of many proteolytic enzymes, which have been shown to play a role in HR [39, 75]. It has been recently shown that GSDM pores induce calcium influx, which modulates pore opening/closing kinetics through a phospholipid-mediated feedback mechanism [76].

(a) CNL resistosome-initiated cell death

**Fig. 2:**
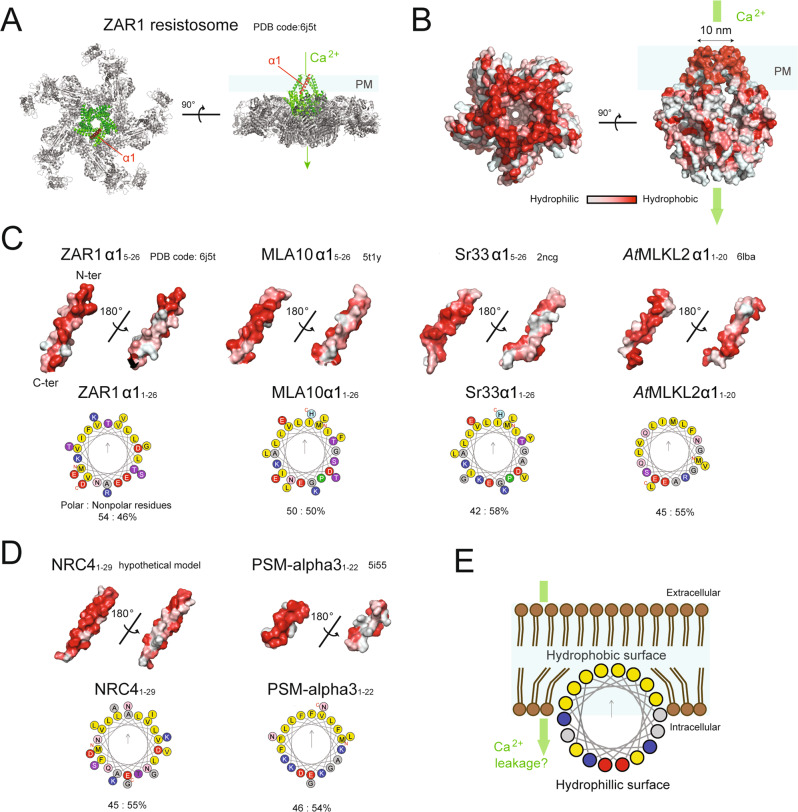
An amphipathic feature of cell death-inducing proteins compromises cell membrane integrity. **A** The pentameric ZAR1 resistosome of *Arabidopsis thaliana* that functions as a Ca^2+^ permeable channel. The solvent exposed funnel-shaped structure constituted by the N-terminal coiled-coil domain and the first helix (α1) of a ZAR1 protomer are highlighted in green and red, respectively. **B** Surface hydrophobicity of the pentameric structure of N-terminal coiled-coil domain of ZAR1. **A**, **B** The presumptive plasma membrane (PM)-inserted region of the ZAR1 resistosome is indicated in each right panel. b)The structures of the first N-terminal helices of selected plant cell death-inducing proteins. **C** The structures of the helices for ZAR1 (*A. thaliana*), MLA10 (barley), Sr33 (wheat), and *At*MLKL2 (*A. thaliana*) are retrieved from the Protein Data Bank. **D** The hypothetical structure of the N-terminal part of NRC4 (Tomato) and the solved structure of PSM-alpha3 (*Staphylococcus aureus*). These two short peptides are able to cause lytic cell death or liposome rupture. **C**, **D** Wheel projections of the α‐helices were built using the server Heliquest (https://heliquest.ipmc.cnrs.fr/). The gray arrows point toward the hydrophobic face of the α‐helices. The hypothetical structure for NRC4 was generated by using the server RaptorX (http://raptorx.uchicago.edu/). **B**–**D** Surface hydrophobicity of helices are visualised by PyMOL using the script “Color h” https://pymolwiki.org/index.php/Color_h. **E** A hypothetical model for the amphipathic helical domain-induced membrane rupture. In this model, hydrophilic surfaces of helical domains are buried in the acyl chains of the phospholipid bilayer that compromises integrity of cell membrane. The cartoon representation is adapted from [119]. A partial membrane rupture could cause ion influxes from extracellular spaces, which might activate cell death program as seen during ferroptosis [108].

The first resistosome described in plants was a pentameric structure containing the activated form of the CNL ZAR1 (HOPZ-ACTIVATED RESISTANCE1) in complex with the pseudokinase PBL2 [72, 77]. Recognition of the cognate bacterial effector AvrAC causes a post-translational modification of PBL2 that results in subsequent activation and oligomerization of ZAR1 into a pentameric wheel structure. In the activated ZAR1 resistosome, the first N-terminal alpha-helixes of the CC domains form a solvent exposed funnel-shaped structure, which constitutes a cation channel (Fig. 2A, B). This structural configuration is required for plasma membrane association and Ca^2+^ influx [72, 77]. The CNL Sr35 also assembles into a resistosome upon direct binding of the fungal effector AvrSr35, forming a structure that is highly similar to the ZAR1 resistosome [71]. Similar to the CNL resistosomes of ZAR1 and Sr35, members of the RNL subclass also exhibit a calcium-permeable unselective cation channel activity [71, 78, 79], likely contributing to the aforementioned long-lasting Ca^2+^ influx. The elevation of cytosolic Ca^2+^ is essential for HR execution, since chemically blocking calcium channels prevents HR cell death [80]. It has been shown that cations other than Ca^2+^ can be transported through resistosome channels [79], indicating that other cations could be important for HR.

(b) TNL resistosome-initiated cell death

Some TNLs assemble into a tetrameric resistosome upon ligand binding, in which two pairs of TIR homodimers function as a nicotinamide adenine dinucleotide (NAD^+^) cleaving enzyme (NADase) [81, 82]. The plant genome encodes a number of TIR-only proteins, which lack some of the typical domains present in TNL proteins (e.g., NB-ARC, LRR domains) [67, 83]. These TIR-only proteins and artificially truncated TIR domains of TNLs can be assembled into filament-like structures that produce 2′,3′-cAMP/cGMP by hydrolysing DNA or RNA [84]. As HR caused by TNLs and TIR-only proteins genetically requires RNLs [85, 68], some of products of the TIR enzymes are anticipated to act as signalling molecule(s) to facilitate the formation of RNL-resistosomes. Recently identified TIR products, 2’-(5”-phosphoribosyl)-5’-adenosine mono-/di-phosphate (pRib-AMP/ADP) [86], and ADP-ribosylated ADPR (di-ADPR) or (ADP-ribosylated ATP or ADPr-ATP) [87] are proposed to be perceived by EDS1-PAD4 and EDS1-SAG101 receptor complexes, respectively, for the formation of RNL-resistosomes [86, 87]. Plant pathogens have evolved effectors to manipulate both precursors and products of TIR-enzymes. For example, an effector of *Xanthomonas* phosphorylates NAD^+^ [88] and effectors from oomycete and bacterial pathogens hydrolyse 2′,3′-cAMP/cGMP but not its regioisomer 3′,5′-cAMP/cGMP [84, 89] emphasising the importance of the TIR-catalysed products in plant disease resistance.

### MLKL-mediated necroptosis

In animals, necroptosis has been defined as a form of regulated necrotic cell death induced by receptor interacting protein kinase 3 (RIPK3) and its substrate mixed lineage kinase-like (MLKL) [90]. RIPK3 is activated by upstream pathways via RIP homotypic interaction motif (RHIM)-dependent protein-protein interactions with the three additional proteins in the mammalian genome that contain conserved RHIMs, namely RIPK1, TRIF and ZBP1/DAI [90, 91]. RIPK1 links RIPK3 to death receptor (DR) signalling: TRIF mediates RIPK3 activation downstream of TLR3 and TLR4, whilst ZBP1/DAI mediates RIPK3 activation in response to certain viruses [91] and endogenous Z-form nucleic acid [92]. Importantly, caspase-8 inhibits RIPK3 activation and the induction of necroptosis by mechanisms that likely involve cleavage of RIPK1/RIPK3 complex components. Necroptosis execution requires the RIPK3-dependent phosphorylation of MLKL. Phosphorylated MLKL induces plasma membrane damage via incompletely characterized mechanisms [91, 93].

Pathogens utilize a plethora of mechanisms to inhibit necroptosis. Several bacterial peptidases expressed by EPEC and Shigella degrade components of necroptotic machinery [94, 95]. However, interference of necroptosis seems to be a common pathogenic strategy of viral pathogens. Viral inhibitor of RIP activation (vIRA) proteins of MCMV (M45) can interact with TRIF, ZBP1, RIPK1, and RIPK3 via RHIM homotypic interaction to impair necroptosis [96]. Unlike MCMV, human CMV (HCMV) ortholog of M45, UL45, does not contain a RHIM domain but efficiently inhibits necroptosis by acting downstream of RIPK3 and MLKL [97]. Cowpox virus expresses viral inducer of RIPK3 degradation (vIRD) triggering K48-linked ubiquitylation of RIPK3 and its proteasomal degradation [98]. Other poxviruses express viral MLKL-like proteins (vMLKL) and inhibit necroptosis by sequestering RIPK3 [99].

In view of the fact that caspase-8 is the central inhibitor of necroptosis, necroptosis has been frequently considered to have evolved as a “back-up” cell death mechanism when death receptor activation fails to kill an infected cell via apoptosis. Intriguingly, poxovirus crmA is able to preferentially inhibit caspase-8-mediated apoptosis without markedly altering caspase-8-mediated inhibition of necroptosis [100]. Herpes simplex virus (HSV)-1 and HSV-2 inhibit apoptosis via direct binding of caspase-8 by the large subunit (R1) of ribonucleotide reductase (RR). HSV-1 and HSV-2 R1 proteins (ICP6 and ICP10, respectively) also prevent necroptosis by inhibiting the interaction between RIPK1 and RIPK3 [101].

MLKLs contain a characteristic N-terminal four-helix bundle structure called the HeLo domain. This domain, named after fungal Het and LopB proteins, is commonly present in a number of cell death-inducing proteins in animals, fungi and plants [68–70, 102, 103]. In fungi, Het-S or Het-s (Heterokaryon incompatibility protein S/s) trigger hyphal death in heterokaryon incompatibility [69]. In plants, the N-terminal RPW8-like CC domains in RNLs (i.e., ADR1 and NRG1 family) are structurally similar to the HeLo domain [69, 78] and the N-terminal domains alone of the ADR1 and NRG1 are sufficient to cause HR cell death [104].

A conserved protein family across seed plants that structurally resembles animal MLKL was recently discovered [70]. Experiments using combinatorial mutants of the three *Arabidopsis MLKLs* (*AtMLKLs*) indicate that they act redundantly in conferring disease resistance mediated by TNLs but not CNLs [70]. Considering that the HeLo domain-containing RNLs are also required for TNL-mediated HR and immunity [68], it may be surmised that two distinctive HeLo domain-containing protein families, namely plant MLKL and RNL families, cooperatively participate in TNL-mediated immunity. Furthermore, the mobility of *At*MLKLs on microtubules is linked to their immune output [70]. This is interesting, considering the fact that microtubules are attributed to the translocation of animal MLKLs from cytoplasmic necrosomes to the plasma membrane [105].Taken together, an analogous biochemical mode of action for plant and animal MLKL-mediated cell death and immunity is anticipated.

### Iron-dependent lytic cell death

Ferroptosis is an iron-dependent, oxidative form of necrotic cell death that does not share genetic similarities with apoptosis, pyroptosis or necroptosis. In animals, ferroptosis can be triggered by depleting the cell of the amino acid cysteine, or by inhibiting phospholipid hydroperoxidase glutathione peroxidase 4 (GPX4) [106]. In contrast to apoptosis, pyroptosis and necroptosis, ferroptotic cell death does not appear to involve molecularly defined cascades of events, but represents a distinct cellular death outcome involving iron-dependent peroxidation of lipids associated with plasma membrane damage [107]. During ferroptosis, a partial membrane rupture could cause ion influxes from extracellular spaces, which might activate further downstream cell death programme [108].

Lipid peroxidation has been long known to be involved in the pathogenesis of diverse infectious diseases, although knowledge about the role of ferroptosis in previous data dealing with lipid peroxidation during the course of infection is lacking. In particular, ROS are frequently formed after viral infections and an imbalance in cellular redox responses has been viewed as one the of the drivers of virus-induced inflammatory destruction of a tissue [109]. Therefore, one can speculate that oxidative stress, lipid peroxidation and ferroptosis can participate in various pathological states of viral infection. In line with this notion, T cell lipid peroxidation and ferroptosis were shown to prevent immunity against Choriomeningitis virus [110]. Similar to viral infection, a few bacterial pathogens have been associated with ferroptosis of the host cell. *M.*  *tuberculosis* has been characterized as a trigger of pathological ferroptosis in macrophages [111]. *P. aeruginosa* utilizes host polyunsaturated phosphatidylethanolamines to trigger theft-ferroptosis in bronchial epithelium [112]. Whether and how ferroptosis interferes and impacts on the course of host–pathogen interaction in animals will constitute future experimental challenges.

In plants, a ferroptosis-like process has been shown to take place in rice upon NLR-mediated recognition of the fungal pathogen *Magnaporthe oryzae* [113]. In this study, ferric ions (Fe^3+^) and ROS were shown to focally accumulate in cells undergoing HR cell death and neighbouring areas. Importantly, the ferroptosis inhibitors deferoxamine and ferrostatin-1 attenuated HR levels. The degree of conservation of ferroptosis between plants and animals and how extended is this type of cell death within the green lineages remain to be determined.

### Loss of plasma membrane integrity in plant and animal cell death

It is becoming increasingly clear that a transient or permanent loss of endomembrane integrity is a key step of many forms of cell death both in animals (pyroptosis, necroptosis, ferroptosis, apoptosis) and plants (HR). In the case of animal cell death, plasma membrane rupture and catastrophic cell lysis are facilitated by the transmembrane protein Ninjurin-1 (NINJ1), upon oligomerization of its amphiphatic extracellular α-helix [114]. An amphiphatic α-helix carries both but segregated hydrophilic and hydrophobic surfaces, a common feature of cation channel-forming pores or pore-forming proteins/toxins [72, 79, 93] (Fig. 2).

In plants, an amphiphatic helical domain is located at the N-termini of many CNLs -including ZAR1, MLA10, Sr33 [72, 115, 116] and they share the “MADA” motif named after the consensus MADAxVSFxVxKLxxLLxxEx [117] (Fig. 2). Although this motif could be attributed to their cation channel functions associated with resistosomes, overexpression of the N-terminal part of NRC containing the “MADA” motif is sufficient to cause HR-like cell death [117]. This is reminiscent of lytic cell death caused by amphiphatic PSM-alpha3 (*Staphylococcus aureus*) [118]. Such an amphipathic feature is also present in the HeLo-helices of mouse, human, and plant MLKLs [70, 93] (Fig. 2C), although how MLKLs induce plasma membrane permeabilization is not well established [70, 91, 93].

How does the amphipathic helical domain cause membrane disruption? Hydrophilic surfaces of the amphipathic helical domain could be buried inside the acyl chains of the phospholipid bilayer of the plasma membrane, which would partially compromise integrity of cell membrane like a surfactant [119] (Fig. 2E). Experimental evidence suggests that oligomerization of amphipathic helical proteins enchases or are required for their cell lytic activities [114, 117, 118]. Partial membrane damage leading to formation of a transient pore can cause ion fluxes, which might activate a cell death programme as described in the case of ferroptosis [108] (Fig. 2E). This model suggests that cell death can be initiated by a range of proteins carrying an amphipathic helical domain, although they may not form well-organised pores or channels and this model might apply to the MLKL-induced plasma membrane permeabilization as previously proposed [93].

### Signalling molecules released from dying cells

**Fig. 3:**
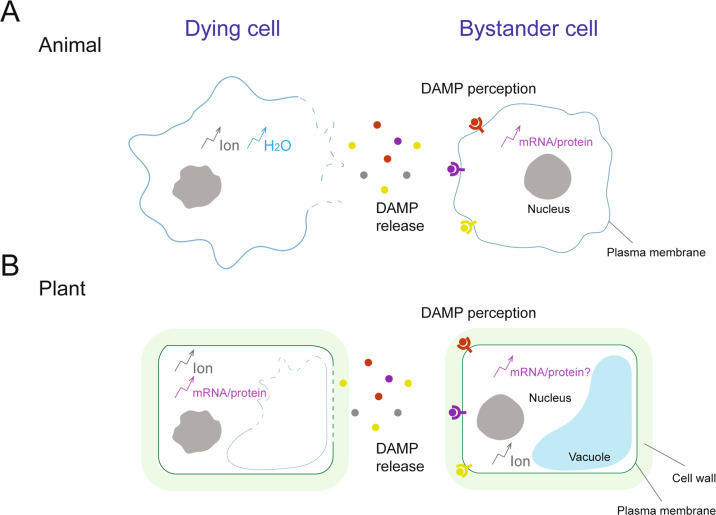
Communication between cells during immunogenic cell death. **A** In animals, activation of host defence systems can initiate a range of genetically encoded cell death, including gasdermin-induced pyroptosis and MLKL-induced necroptosis. Some pathogenic microbes secrete toxins to manipulate host immune systems. Ion- and water- fluxes are commonly seen during cell death progression. The water entry into cells results in cell swelling and blebbing and cell rupture is finally executed by Ninjurin-1 (NINJ1) [114]. Cell rupture facilitates the release of damage-associated molecular patterns (DAMPs), which stimulate inflammatory responses of neighbouring cells. **B** Like in animals, plant cell death plays fundamental roles in immunity. The hypersensitive response (HR) is a rapid localized cell death at pathogen invading sites mostly mediated by intracellular NLR immune receptors. Unlike animal cell death, rigid cell walls prevent cell swelling and blebbing. Cell rupture facilitates the release of damage-associated molecular patterns (DAMPs), which stimulate immune responses of neighbouring cells.

Conceptually, lytic cell death including pyroptosis, necroptosis and ferroptosis in animals has been originally considered to cause an uncontrolled release of cellular contents and exposure of DAMPs, since the cell boundary is lost due to membrane rupture (Fig. 3A). It is however increasingly evident that different modes of regulated necrosis provoke the release of a distinct battery of DAMPs. Accordingly, HMGB1 released by pyroptosis is hyperacetylated, which is not the case when it is released from necrotic or apoptotic cells [120]. Furthermore, in contrast to initial views, apoptotic cells can also release different factors such as nuclear DNA (below 180 bp), ATP (early phase) and HMGB1 (late phase) [121].

The composition and signatures of apoptotic and necroptotic DAMPs might be qualitatively and quantitatively different. In apoptosis, caspases process different factors such as phospholipase A2 (PLA2) and inhibitor of caspase-activated DNase (called ICAD) to induce phagocytosis and to produce small and weak immunostimulatory DNA fragments. In addition, full-length IL-33 is released during necroptosis, but during apoptosis it can undergo caspase-dependent proteolysis into a non-immunogenic form [122]. DAMP release has been significantly less studied in the context of necroptosis. However, as membrane integrity is lost in necroptosis in a fashion similar to necrosis, theoretically, necroptosis also results in the release of DAMPs and other cellular components including, but not limited to, HMGB1, ATP, histones, HSPs, exRNAs, and cfDNA. Pyroptosis is typically known for the release of IL-1ß and IL-18, though some other DAMPs such as HMGB1, ATP and cfDNA can be released by cells undergoing pyroptosis. Although it is a relatively new concept and less has been elucidated yet, HMGB1 and cfDNA have been regarded to be released by ferroptosis [123].

Plant cells also release a large range of DAMPs or phytocytokines into the surrounding environment upon pathogen perception, to alert neighbouring cells and activate immunity, including peptides, nucleotides, sugars and amino acids [124–126] (Fig. 3B). However, it remains to be established how dying and dead cells contribute to the release of signalling molecules during plant-pathogen interactions. A hint for such a mechanism could be inferred from plant responses to wounding, oxidative, and biotic stresses [127, 128]. Upon mechanical damage, the Ca^2+^-activated metacaspase *At*MC4 cleaves a 23-residue peptide called PEP1 from its precursor PROPEP1. As PROPEP1 and PEP1 do not contain canonical signal sequences for extracellular secretion [127], plasma membrane damage during cell death can promote the release of the peptide (Fig. 3B). The released PEP1 is recognized by its cognate plasma membrane localised receptor [129]. Other extracellularly released DAMPs upon membrane damage may include extracellular ATP [130] and oligogalacturonides, a constituent of the plant cell wall [131]. Collectively, these examples provide a mechanistic clue for how DAMPs that lack the signal sequences for secretion could be released into extracellular spaces upon membrane disintegration. However, this field of research is still in its infancy and these molecules could as well be released by alternative secretion mechanisms that are so far unknown.

### Calcium entry as a danger signal driving membrane repair to counteract cell death machinery

While a long-lasting Ca^2+^ influx would facilitate HR cell death, Ca^2+^ entry or leakage (Fig. 2) caused by plasma membrane damage or pore forming proteins can activate membrane repair machinery [132, 133]. Therefore, the point where membrane damage exceeds the capacity for membrane repair would define “Point-of-no-return” of dying cells. In animals, the repair machinery removing lesions from the plasma membrane includes endocytosis, ESCRT-complex mediated shedding, exocytosis-mediated patching and annexin-mediated sealing [132, 133]. Although these processes can seal off lesions of Ca^2+^ entry, failure to prevent Ca^2+^ entry can result in a prolonged Ca^2+^ influx.

In animals, increases in cytosolic Ca^2+^ have long been shown to cause cell death with or without engaging cell death machinery. Our knowledge about the molecular events and physiological relevance of these findings is however still fragmentary. A Ca^2+^-related mechanism was proposed more than two decades ago to explain necrosis incurred in cardiac ischemia and muscular dystrophy by involving phospholipases and proteases, leading to release of free fatty acids and their breakdown products and to degradation of cytoskeletal proteins [134]. Under pathological conditions of cellular cytosolic Ca^2+^ overload, particularly in association with oxidative stress, alteration of mitochondrial Ca^2+^ uptake causes cellular damage and necrosis. Long-lasting opening of mitochondrial permeability transition pore (PTP) under high Ca^2+^ concentrations induces mitochondrial damage and triggers cell death which is accompanied by mitochondrial apoptosis [135], the biologic role of which remain elusive. The prolonged and elevated cytoplasmic Ca^2+^ concentration was shown to cause irreversible formation of ceramide platforms within the plasma membrane, which is proposed to induce a proximity of FAS (CD95) receptors to fully activate caspase-8 [136]. The generation of ceramide-enriched membrane is irreversible, thereby defining the ‘Point-of-no-return” of dying cells [137]. Mutations in the gene encoding NLRP3 cause a spectrum of autoinflammatory diseases known as cryopyrin-associated periodic syndromes (CAPS). Increased extracellular Ca^2+^ concentrations trigger activation of the NLRP3 inflammasome in monocytes through Ca^2+^-sensing receptor (CaSR) which causes inflammation in human cryopyrin-associated periodic syndrome (CAPS) [138, 139].

In plants, the prolonged and elevated cytoplasmic Ca^2+^ concentration is tightly associated with HR [140]. Whether plants have genetically encoded-machinery to sabotage membrane repair machinery or to detect a sustained Ca^2+^ influx for final cell death executioner activation during HR remains to be determined.

### Concluding remarks

Traditional studies focusing on the role of cell death and its regulation in plants and animals mainly considered caspase-induced apoptosis as the only regulated cell death process and viewed death as the ultimate end point. Those initial studies failed to identify shared molecular features of cell death among the two kingdoms. In contrast to apoptosis, plant cell death is usually accompanied by membrane disintegration and the characteristic features of apoptosis are not induced in plants, including caspase activation. The recent discovery of molecularly controlled pathways of lytic cell death in animals has redefined necrosis as a regulated cell death process. Accumulating evidence in the past decades identified regulated necrosis as a powerful trigger of inflammation, indicating that the mediators of cell death additionally control inflammatory signalling. In particular caspases, originally described as engines of apoptosis can, however, independently control lytic cell death and membrane rupture. Accordingly, the lack of caspase-8 causes lethality in mice because of its failure to inhibit necroptosis, but not because of its failure to induce apoptosis.

Membrane pore formation has indeed emerged as a major shared characteristic between plant and animal immunogenic cell death. This has become particularly intriguing with i) the discovery of plant resistosomes comprising activated NLRs that can directly form pores in the membrane that allow Ca^2+^ influx and ii) the characterization of functional/structural homologs of animal MLKLs in plants [70], revealing necroptosis as a relevant lytic cell death option in plants. Besides, the discovery of GSDM-like pore-forming proteins outside of mammals and vertebrates indicates that the GSDM family is of extremely old evolutionary origin and likely a core-component of immunity in divergent eukaryotic and prokaryotic taxa [141]. It is important to note that some recently described GSDM-like proteins in fungi show remarkable structural similarity to animal proteins [142]. One important task for future experimental work will be to identify and characterize plant GSDM-like proteins. This may pave the way for further identification of proteases controlling GSDM function and membrane integrity in plants.
